# Surface terminations control charge transfer from bulk to surface states in topological insulators

**DOI:** 10.1038/s41598-024-61172-6

**Published:** 2024-05-08

**Authors:** Keiki Fukumoto, Seunghee Lee, Shin-ichi Adachi, Yuta Suzuki, Koichi Kusakabe, Rikuto Yamamoto, Motoharu Kitatani, Kunio Ishida, Yoshinori Nakagawa, Michael Merkel, Daisuke Shiga, Hiroshi Kumigashira

**Affiliations:** 1 High energy accelerator research organization (KEK), 1-1 Oho, Tsukuba, Ibaraki 305-0801 Japan; 2 The Graduate University for Advanced Studies (SOKENDAI), Hayama, Kanagawa 240-0193 Japan; 3 University of Hyogo, 3-2-1 Kouto, Kamigori-cho, Ako-gun, Hyogo, 678-1297 Japan; 4 Utsunomiya University, 7-1-2 Yoto, Utsunomiya, Tochigi 321-8585 Japan; 5 Nichia Corporation, 491 Oka, Kaminaka, Anan, Tokushima 774-8601 Japan; 6 FOCUS GmbH, Neukirchner Str.2, 65510 Huenstetten, Germany; 7 Tohoku University, Katahira 2-1-1, Aoba-ku, Sendai, Miyagi 980-8577 Japan

**Keywords:** Topological insulator, Dirac states, Ultrafast, Photoelectron spectroscopy, Microscope, Materials science, Condensed-matter physics

## Abstract

Topological insulators (TI) hold significant potential for various electronic and optoelectronic devices that rely on the Dirac surface state (DSS), including spintronic and thermoelectric devices, as well as terahertz detectors. The behavior of electrons within the DSS plays a pivotal role in the performance of such devices. It is expected that DSS appear on a surface of three dimensional(3D) TI by mechanical exfoliation. However, it is not always the case that the surface terminating atomic configuration and corresponding band structures are homogeneous. In order to investigate the impact of surface terminating atomic configurations on electron dynamics, we meticulously examined the electron dynamics at the exfoliated surface of a crystalline 3D TI (Bi_2_Se_3_) with time, space, and energy resolutions. Based on our comprehensive band structure calculations, we found that on one of the Se-terminated surfaces, DSS is located within the bulk band gap, with no other surface states manifesting within this region. On this particular surface, photoexcited electrons within the conduction band effectively relax towards DSS and tend to linger at the Dirac point for extended periods of time. It is worth emphasizing that these distinct characteristics of DSS are exclusively observed on this particular surface.

A three-dimensional topological insulator (3D TI) is a material exhibiting bulk insulating behavior, yet possessing dissipationless, metallic surface states referred to as the Dirac states (DS)^1,2^. Due to the exceptional transport properties of massless electrons in these states, TIs are being developed for a wide range of applications including fast transistors^3–5^, atomically thin transparent electrodes^6^, terahertz light detectors^7^, and generators^8,9^. The performance of such devices is heavily dependent on the dynamic properties of Dirac electrons. For example, spintronic devices require a higher spin polarization and longer lifetime are required^10,11^.

The single crystal Bi_2_Se_3_ is a widely studied and prototypical 3D TI^2^; it is composed of quintuple layers (QLs) held together by weak van der Waals interactions. Each QL is comprised of five atomic sheets made of either Se or Bi, which are covalently bonded together (see Fig. 1a). The surfaces of crystalline Bi_2_Se_3_ can be easily prepared through exfoliation, and the crystal is expected to peel off between QLs, resulting in a Se-terminated surface (S1 in Fig. 1) at the topmost surface of the structure. However, previous reports have suggested and our atomic force microscopy experiments (Fig. S5 and S6) confirmed that this is not always the case, and the crystal can be cleaved within a QL, resulting in locally different surface terminations. Surface science techniques^12–17^ and theoretical works^18–21^ have shown that the density of states (DOS) of such surfaces is dependent on surface terminations, specifically the energy level of the Dirac point (DP) within the bulk band gap and the distribution of surface charge. Additionally, inhomogeneities in the density of surface states (SSs) have been predicted based on the results of infrared spectroscopy^22,23^, terahertz Kerr rotation measurements^24,25^, electron transport measurements^26^, and angle-resolved photoemission spectroscopy (ARPES) using a sub-mm beam spot^27^.

**Figure 1:**
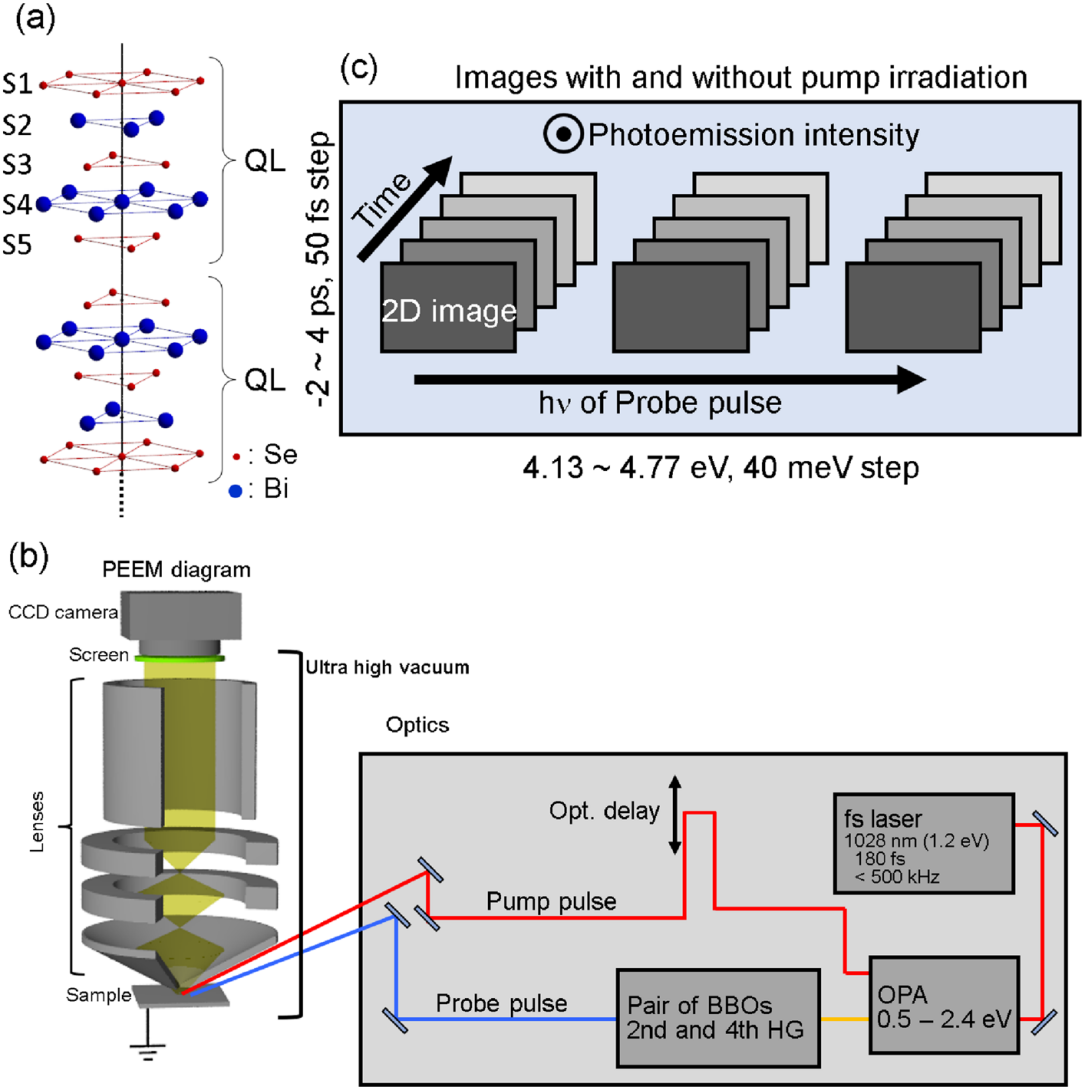
(**a**) Atomic arrangement of Bi_2_Se_3_. The five atomic layers are termed S1, S2, S3, S4, and S5, in this article. (**b**) Schematic of the CDC-PEEM setup. (**c**) Dataset for the CDC-PEEM experiments.

Harnessing the potential of TIs for the aforementioned applications requires a thorough understanding of the dynamics of DS electrons depending on different surface terminations. The dynamics of photogenerated electrons have been extensively studied using time-resolved ARPES (TR-ARPES)^28–31^. The bulk conduction band (BCB) has been reported to act as a reservoir for photogenerated electrons, supplying them to DSs over a longer timescale than that resulting from energy relaxation alone. Transient reflectivity experiments have also verified the transfer of charge from BCB to DS^32–35^; however, these methods lack the spatial resolution necessary to extract dynamics reflecting surface inhomogeneity.

In this article, we present observations of photogenerated electron dynamics at the surface of a typical three-dimensional topological insulator, Bi_2_Se_3_, using photoemission electron microscopy with femtosecond laser pulses as the excitation source (fs-PEEM). fs-PEEMs is widely utilized by virture of its high temporal and spatial resolutions, and can also provide energy resolution information using an energy filter capable of determining the photoelectrons’ kinetic energy^36,37^. However, we demonstrated that efficiently inducing photoemission from CB in semiconductors, in which the electron density is several orders of magnitude lower than that of the valence band (VB), requires precise adjustment of photon energy in the vicinity of the ionization energy of the conduction electrons. Acquiring photon energy-dependent PEEM images enabled us to map the energy levels. Because this method is sensitive to electron density, we term it charge density contrast photoemission electron microscopy (CDC-PEEM). Schematic of the CDC-PEEM setup and the summary of the dataset acquired in this study are displayed in Fig. 1b,c, respectively. Those details are in Supplementary Information (SI) and can be also found elsewhere^38–42^. By combining CDC-PEEM with first principles calculations, we discovered that the spatial inhomogeneity of photoemission (PE) intensity is the result of different surface terminations and leads to distinct electron dynamics.

## Results

### Photon energy dependent PEEM images

**Figure 2:**
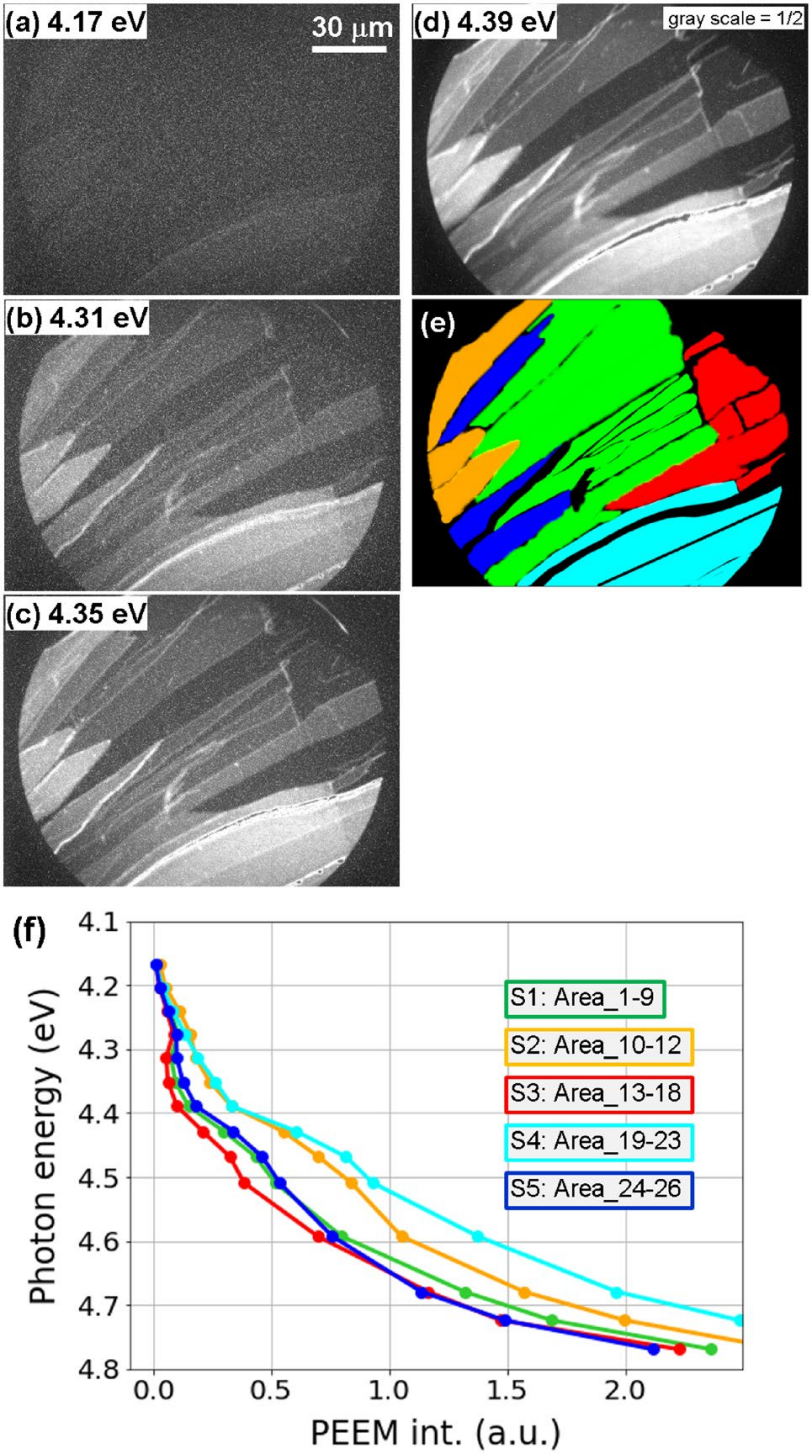
(**a**–**d**) PEEM images showing a Bi_2_Se_3_ surface with 4.17, 4.31, 4.35, and 4.39 eV excitation sources. (**e**) Domains shown in (**a**–**d**) after being classified and colored. (**f**) Spectra obtained from all 26 regions averaged within the same colored regions as shown in (**e**) and Fig. S1.

Figure 2(a–d) show PEEM images acquired with probe pulse energies of 4.35, 4.31, 4.35, and 4.39 eV , respectively, without pump pulse irradiation. The images display micrometer-scale domains, which are identified by their distinct PE intensities and shown in Fig. 2e. It should be noted here that since the spatial resolution of CDC-PEEM is 50 nm, it is not possible to detect even if there are nanometer-high steps of about 1 QL within a domain. Furthermore, PE intensity does not vary with different terrace heights. PE intensity obtained in the CDC-PEEM experiments has the characteristic that it depends only on the density of electrons and the depth of energy level from the vacuum level. In other words, the domain observed here reflects the electronic state, not the morphology. Domains are numbered as in Fig. S1. Comprehensive data analysis and the results of first principles calculations enable the regions to be classified into five categories, as illustrated with distinct colors in Fig. 2e. These regions are determined to be the S1, S2, S3, S4, and S5 atomic layer terminations, respectively, corresponding to regions 1–9, 10–12, 13–18, 19–22, and 23–26 in Fig. S1.

### Locally different photoemission spectra

The spectrum within each of the 26 domains is inferred from the photon energy-dependent PEEM images by averaging the PE intensity within the colored regions. Subsequently, the spectra are averaged across the same colored regions, and are represented with their respective colors in Fig. 2f. The PE thresholds are roughly 4.15 eV for all five spectra shown in Fig. 2f, which align with the Fermi energy (E_F_), and denote the BCB of n-type semiconductors. This concurs with previously reported observations for n-type Bi_2_Se_3_ single crystals^43–45^.

### The first principle calculations to reproduce the spectra

**Figure 3:**
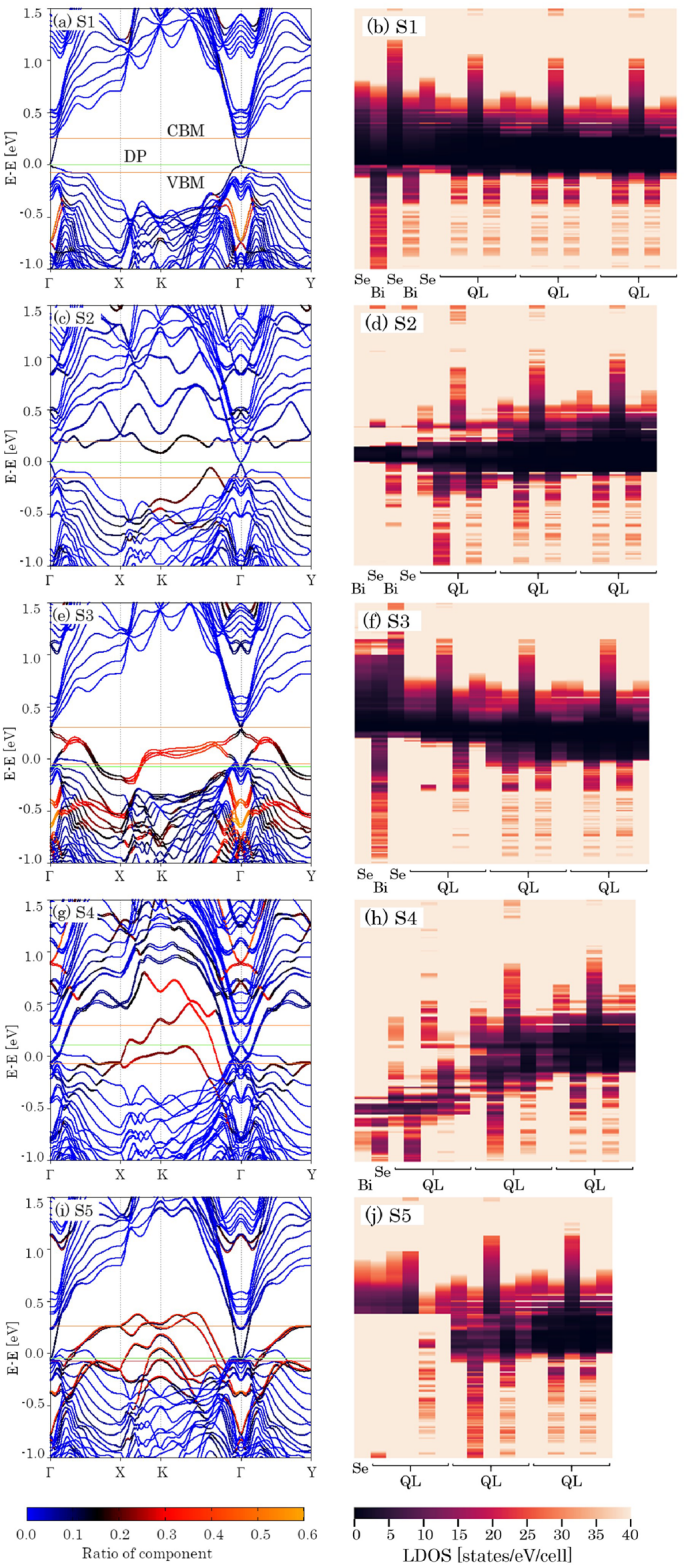
Electronic band structures (**a**, **c**, **e**, **g**, and **i**) and surface localized density of states (**b**, **d**, **f**, **h**, and **j**) for different surface terminations.

To reproduce the spectra in Fig. 2f, we performed the first principle calculations. The calculated bulk and surface band structures of all five surface terminations are shown in the left column of Fig. 3. The blue curves indicate bulk. The surface bands are shown in red and orange depending on the intensity of the projection on the topmost surface (see details at the bottom-left of Fig. 3). The conduction band minimum (CBM) and valence band maximum (VBM) are indicated by red horizontal lines and a green line denotes the DP in each panel.

On the S1 surface (Fig. 3a), Dirac cone is clearly seen in the bulk gap, similar to previously published calculations^46–48^, and experimentally observed structures in Ref.^44^. The surface band appears in a deep part of VB with the exception of the Fermi level such that no contribution from the surface states appears around the Fermi energy except for the Dirac cone states. Owing to their topological protection, Dirac cones also appear at the $\Gamma$ point on the other four surfaces, however, the structures of non-Dirac SSs are different.

The distributions of electron density along the depth axis of the crystal (real-space local density of state: LDOS) were also estimated and are shown in the right column of Fig. 3 for the five studied surfaces; the same vertical axes as shown as in the left panels. The horizontal axis denotes the real space distance from the surface to the fourth QL including the exfoliated QL. The color scale of LDOS is shown at the bottom. The LDOS gap structure appears energetically higher on Se and deeper on Bi. It is visible as a contrast variation within a single QL and is remarkably high on Se in the center of each QL. As the viewpoint approaches the surface, the gap is found to be energetically higher and larger for S1 and S3, whereas it becomes lower and smaller for S2 and S4.

The contribution to LDOS coming from Dirac cones does not present a very prominent in-gap state. This is due to the fact that the Dirac cones exhibit states of relatively slowly-decaying damping waves. Their contribution appears as a thin LDOS hump near the surface. One can see that the bulk gaps are clearly open, in the fourth QL. For the S1 surface, since the DPs are located near the bulk valence band (BVB), the bottom of VBs appear to be elevated relative to the surface (Fig. 3b). In contrast, the contribution from surface localized states appearing as in-gap states above and below the gap is superimposed on S2 and S4. As the result, the gap size decreases. In S3 and S5, the surface localized states are also clearly visible in VB where the surface bands are colored red. The surface states cause increases in LDOS near the surface in Fig. 3f,j. But CB appears to be largely elevated along the energy axis, and consequently, its gap structure remains near the surface.

The orange and cyan curves in Fig. 2f are assigned to the S2 and S4 surfaces respectively; these exhibit a monotonic increase in intensity with increasing photon energy. Our band structure calculations reproduce these results, indicating that the Bi layer is situated at the top (S2 and S4 surfaces), the prominent SSs occur as high as DP between the *K* and $\Gamma$ points, and surface bands appear in the upper parts of the gap associated with S2 surface (Fig. 3c). Non-Dirac SSs are largely spread within the forbidden band in the *k*-space of the S4 surface (Fig. 3g). As a results, the forbidden band becomes narrower closer to the surface, as illustrated in Fig. 3c,g, and two thresholds originating from BCB and BVB do not appear in the spectra. In contrast, the spectra for the remaining three plots display a plateau up to 4.4 eV, because the bulk band gap is clearly open at the surface as seen in Fig. 3a,e,i. Then at 4.4 eV, the photon energy is sufficiently large to access the BVB, and the photoemossion intensity begins to increase again. The bulk band gap is estimated by our simulations is 0.36 eV (Fig. 3), consistent with the reported values observed by angle-resolved PES^43–45^.

### Time, space, and energy dependent experiments

The time- and energy-dependent PE intensities for all 26 regions are displayed in Fig. S2 (Supplemental Material). The vertical axis represents the PE intensity with pump pulse irradiation divided by one without pump pulse irradiation. The two horizontal axes denote time and probe pulse energy, respectively. Selected representative plots were chosen from the five regions (Areas 1, 10, 13, 19 and 24) with probe energies of 4.17, 4.31, 4.39, and 4.77 eV, and are displayed in the upper row of Fig. S3, where the vertical axis is normalized from 0 to 1. Although the spectral shapes are similar for the S2 and S4 surfaces, PE intensities below 4.2 eV in Fig. S3(i) are more pronounced than in Fig. S3g. Our simulation concludes that the DOS of S4 above the Fermi level is higher than that shown in S2 (Fig. 3).

### Electron transfer from bulk to Dirac surface states

**Figure 4:**
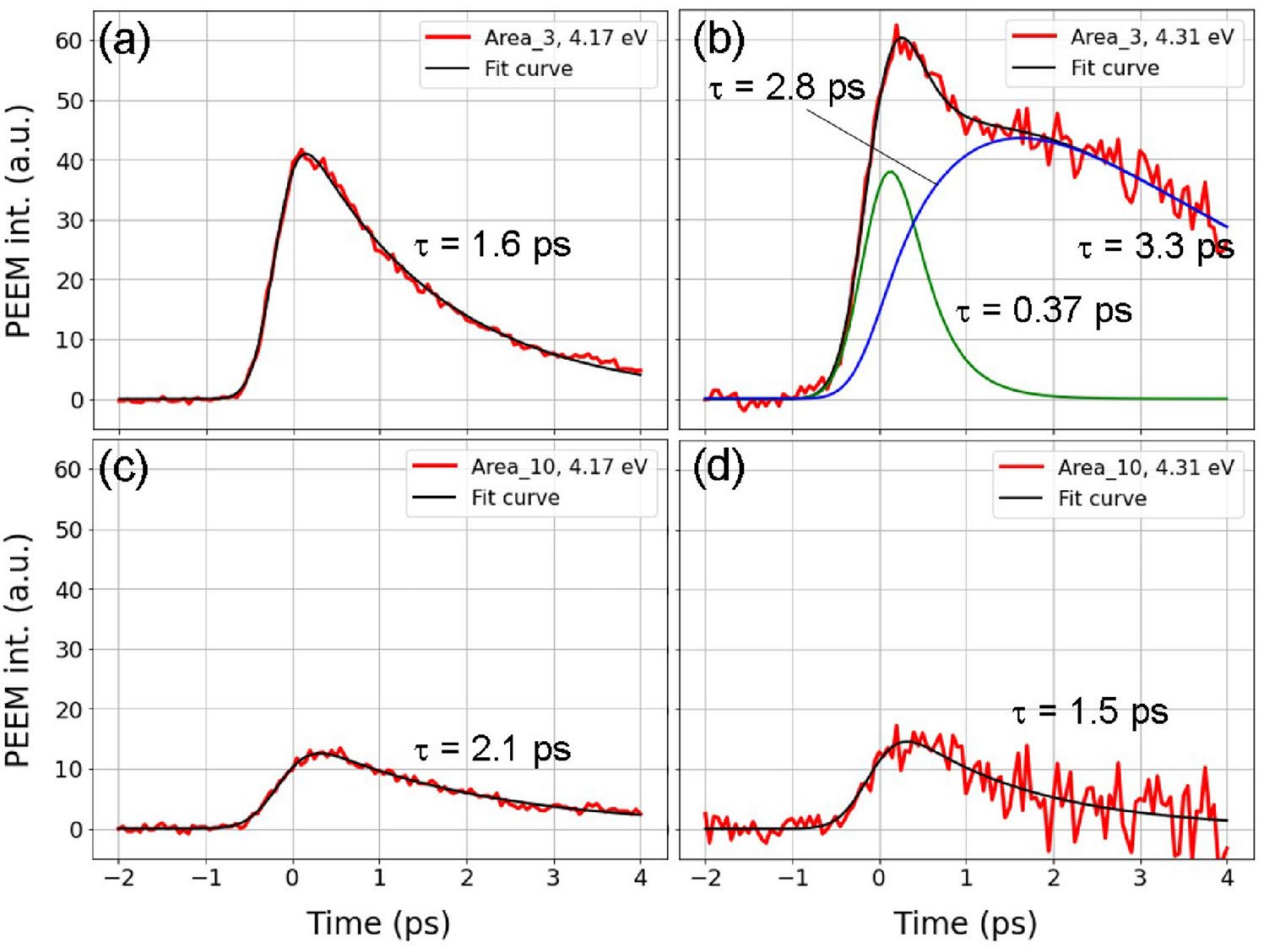
(**a**, **b**) Time dependent PE intensities for Area 3 with probe energies of 4.17 and 4.31 eV. (**c**, **d**) Time dependent PE intensities for Area 10 with probe energies of 4.17 and 4.31 eV.

Most of the time-dependent intensity profiles shown in Fig. S2 could be fitted using a single exponential decay function convoluted by a Gauss function with a full width at half maximum of 600 fs. The decay time constants are shorter than 2 ps, indicating that only simple energy relaxation occurs. However, there are exceptions; for example, on the S1 and S3 surfaces at a probe energy of 4.31 eV, where the energy is just sufficient to photoemit electrons from DPs, as shown with orange plots in Fig. 3a and c. The photoelectron intensities do not decay monotonically but increase just after pump pulse irradiation with a certain time constant. The dynamics of the S1 (region 3) and S2 (region 10) surfaces are compared in Fig. 4, which also shows extracts from Fig. S2. Time profiles on the S1 surface at a photon energy of 4.17 eV are shown as red plots in Fig. 4a and the fitting curve is shown in black. This photon energy was just sufficient to induce photoemission from BCB, but not from other deeper states. The observed decay constant of approximately 1.5 ps denotes electron transfer from BCB to lower states. It is significant that the probe energy that is just sufficient to access DP (4.31 eV) is greatly different from the others, as shown in Fig. 4b. The plot is reproduced by a function

$$\begin{aligned} A1 * \exp {(-t/\tau _1)} + A1 * \exp {(-t/\tau _2) * (1-\exp {(-t/\tau _3)})} \end{aligned}$$

convoluted with the same Gaussian function, where A1 and A2 are amplitudes of each component, *t* is time, and $\tau _1$, $\tau _2$, and $\tau _3$ are time constants. After fast decay with $\tau _1$ = 0.4 ps (green plots), the PE intensity increases with $\tau _2$ = 2.8 ps (blue plots). This indicates that electrons are transferred from BCB to DS. Thereafter, the lifetime ($\tau _3$) observed is longer than the time window of this experiment, consistent with previously observed values on the order of 10 ps^28–30,49^. As described in Ref.^28^, BCB acts as an electron reserve from which SS is filled. A long recombination at DP, as observed in graphene^50^, controls these dynamics. For area 10 (S2 surface), the PE intensity monotonically decreases with time at all photon energies. The 4.17 and 4.31 eV pulses are shown in Fig. 4c,d. As mentioned above, since the SSs almost connect BCB and BVB, the electrons promptly relax without passing through the DP. Note that for the plots shown in Fig. 4, the vertical axis is obtained by subtracting the PEEM images with pump irradiation by those without pump irradiation. This corresponds to the density of the excited electrons.

Time-dependent spectra extracted from Fig. S2 are presented in the lower part of Fig. S3; these are also consistent with our calculations. On the S2 and S4 surfaces, the enhancement of PE signals by electron doping is smaller than for the other three surfaces. As shown in the PEEM images in Fig. 2a–d and the corresponding spectra in Fig. 2f, more electrons are present at the initial state (with gaps that are almost closed at the surfaces). On the other hand for the S1 surface, fewer electrons are in BCB before pumping and a longer electron relaxation time via DP is observed. For the S3 and S5 surfaces, since the gaps are open at the surfaces, the signals corresponding to photogenerated electrons are relatively large. However, SSs spread in the gap, pronounced on the S5 surface, and become pathways for electron relaxation, indicated less influence from DP.

In summary, we developed a CDC-PEEM technique that has high temporal, spatial, and energetic resolutions of 100 fs, 100 nm, and 40 meV, respectively, and used it to investigate the transport properties of photogenerated electrons in a 3D topological insulator, Bi_2_Se_3_. Mechanical exfoliation peels off not only between QLs but also in a QL, and the surface is inhomogeneous at the micrometer scale, featuring terminating atomic sheets. Our first principles calculations show that the surface band structures are different exhibiting surface terminations that explain our experimental observations, locally different photoelectron spectra, and diverse electron dynamics. On one Se terminated surface, termed S1 in this article, DP is located at a lower level in the bulk gap and there are no other surface states in the gap. Consequently, photogenerated electrons relax via DP and have a longer lifetime relative to the other four surfaces.

Charge transfer in two dimensional materials such as graphene and transition metal dichalcogenides is a major issue for the next generation of electronic, photoelectronic, and spintronic devices. CDC-PEEM could be a key for investigating this issue.

## Methods

### Experimental condition of CDC-PEEM measurements

The CDC-PEEM consists of a PEEM system (Focus GmbH) and a femtosecond laser source (Light conversion, Pharos SP). Using an optical parametric amplifier (Light conversion, Orpheus) and nonlinear crystals, photon energy of fs laser pulse can be tuned from 0.5 V to 5.9 eV. The experiments were conducted by incrementally adjusting the delay time between the pump and probe pulses from −2 to 4 ps at 0.05 ps intervals, as well as by incrementally adjusting the photon energy of the probe pulses from 4.13 to 4.77 eV at an interval of approximately 40 meV. At each step, PEEM images were acquired with and without pump pulse irradiation. A photon energy of 1.2 eV pump pulses was utilized to excite electrons, exceeding the bulk band gap of 0.36 eV, as per our calculations and approximately 0.3 eV according to^16,48,51^. The repetition rate of the laser pulses was 20 kHz, and the exposure time for the CCD camera was 10 s. Consequently, 200,000 pulses were used to create a single image. The sizes of the pump and probe pulses on the sample surface exceeded the field of view of the PEEM setup. A schematic of the experimental setup is provided in Fig. 1b, and a summary of the experimental conditions is given in Fig. 1c.

### First principles calculations

The electronic band structures of Bi_2_Se_3_ were obtained according to density-functional-theory calculations using the Kohn-Sham scheme^52^. To introduce spin-orbit coupling, the fully relativistic pseudopotential created by the modified Rappe-Rabe-Kaxiras-Joannopoulos scheme was used^53,54^. The generalized gradient approximation by the Perdew-Burke-Ernzerhof parametrization^55^ with the DFT-D3 correction^56^ was also adopted. The bulk band structure of Bi_2_Se_3_^57^ given by the above-mentioned methods was reproduced. The calculations were done using the Quantum ESPRESSO package^58^. To study the surface band structures S1–S5, symmetric slab models with 6QL or 8QL bulk and surface layers were constructed. Structural optimization was performed using a 10 x 10 x 1 Monkhorst-Pack mesh with plane-wave cutoffs of 55 Ry and 500 Ry for the wave function and the charge density, respectively. Each slab included a vacuum layer whose width was greater than 15 Å.

### Supplementary Information

Supplementary Information.

## Data Availability

The datasets used and/or analysed during the current study available from the corresponding author on reasonable request.
