# Supplementary material for: Surface terminations control charge transfer from bulk to surface states in topological insulators
Source: Sci Rep. 2024 May 8;14:10537. doi: 10.1038/s41598-024-61172-6 (PMC11079079; doi:10.1038/s41598-024-61172-6)
Supplement: Supplementary file 1 — Supplementary Information. [file 41598_2024_61172_MOESM1_ESM.pdf]

Supplementary Information: Surface Terminations Control Charge Transfer from Bulk to Surface States in Topological Insulators

Keiki Fukumoto,* Sunghee Lee, and Shin-ichi Adachi

High energy accelerator research organization (KEK), 1-1 Oho, Tsukuba, Ibaraki 305-0801, Japan

Yuta Suzuki

The Graduate University for Advanced Studies (SOKENDAI), Ibaraki, Japan

Koichi Kusakabe, Rikuto Yamamoto, and Motoharu Kitatani

University of Hyogo, 3-2-1 Kouto, Kamigori-cho, Ako-gun, Hyogo 678-1297, Japan.

Kunio Ishida

Utsunomiya University, 7-1-2 Yoto, Utsunomiya, Tochigi 321-8585, Japan.

Yoshinori Nakagawa

Nichia Corporation, 491 Oka, Kaminaka, Anan, Tokushima 774-8601, Japan

Michael Merkel

FOCUS GmbH, Neukirchner Str.2 65510 Huenstetten, Germany

Daisuke Shiga and Hiroshi Kumigashira

Tohoku University, Katahira 2-1-1, Aoba-ku, Sendai, Miyagi 980-8577, Japan

I. DETAILS OF THE CDC–PEEM SYSTEM

A diagram of the experimental setup of charge density contrast photoemission electron microscopy (CDC–PEEM) is in Fig. 1(b) in the main text. A PEEM system (FOCUS GmbH) is used as a detector, and excitation source is provided by femtosecond laser pulse system (Light Conversion, Pharos SP). Using an optical parametric amplifier (OPA) (Light Conversion, Orpheus) and non-linear optical crystals, the photon energy is continuously tunable covering IR to UV range from 0.5 to 5.9 eV. For the present experiments, 4.13 to 4.77 eV pulses are used to induce photoemission (PE) from the bulk conduction band (BCB), bulk valence band (BVB), and Dirac surface state(DS). The resultant energy resolution of this method is approximately 40 meV. The photon energy of the pump pulses to excite electrons is 1.2 eV. The full width of half maximum of a cross–correlation of the pump and probe pulses is 0.6 ps, which defines the temporal resolution. The spatial resolution is 100 nm on the experimental setup.

Using CDC-PEEM, time, space, and energy dependent experiments were performed. A summary of the dataset acquired in this study is shown in Fig. 1(c). The delay times between pump and probe pulses were in the range -2 to 4 ps at 50 fs steps. At each probe energy and at each delay time, PEEM images were obtained using the probe pulses, with and without pump pulse irradiation. In total, 4114 images were stored. The exposure time for the CCD camera was 10 s for each image. Thus, including wait times, the total measurement time was approximately 12 hours.

The time- and energy-dependent PE intensities for all 26 regions indicated in Fig. 2 and Fig. S1 are displayed in Figure S??. The vertical axis represents the ratio of the PE intensity under pump pulse irradiation to that without pump pulse irradiation, and the two horizontal axes denote the time and the probe pulse energy, respectively, as indicated at the bottom of Fig. S??.

* keiki@post.kek.jp

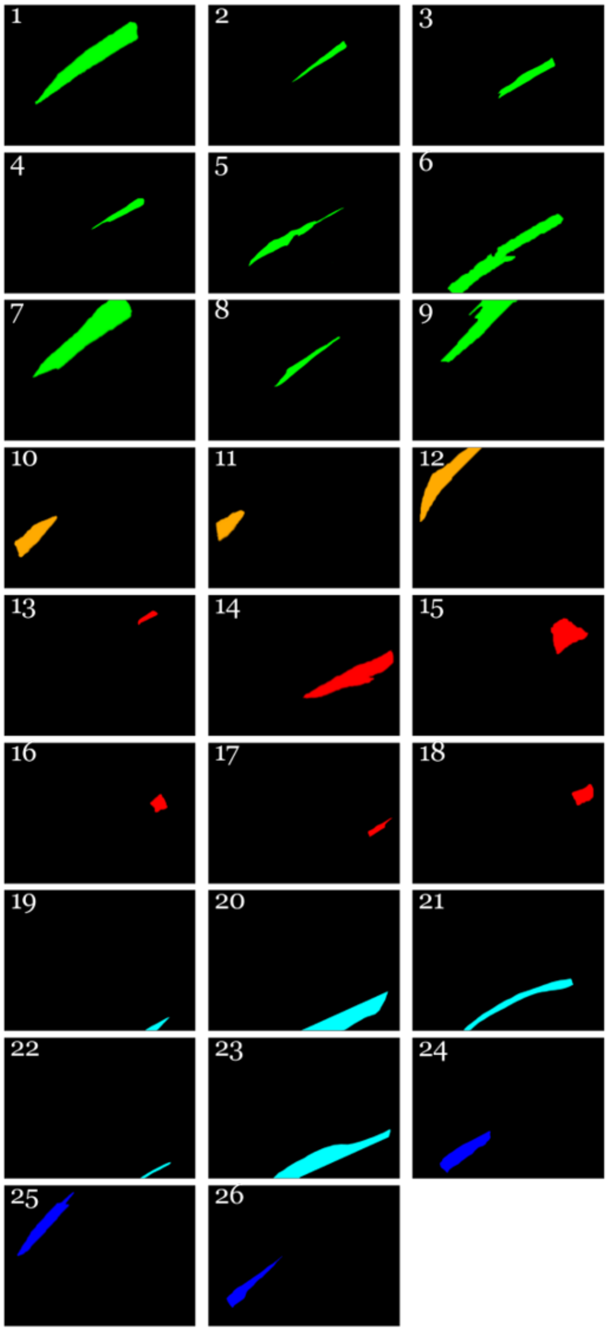

Figure S 1. Observed domains in this study are numbered from 1 to 26.

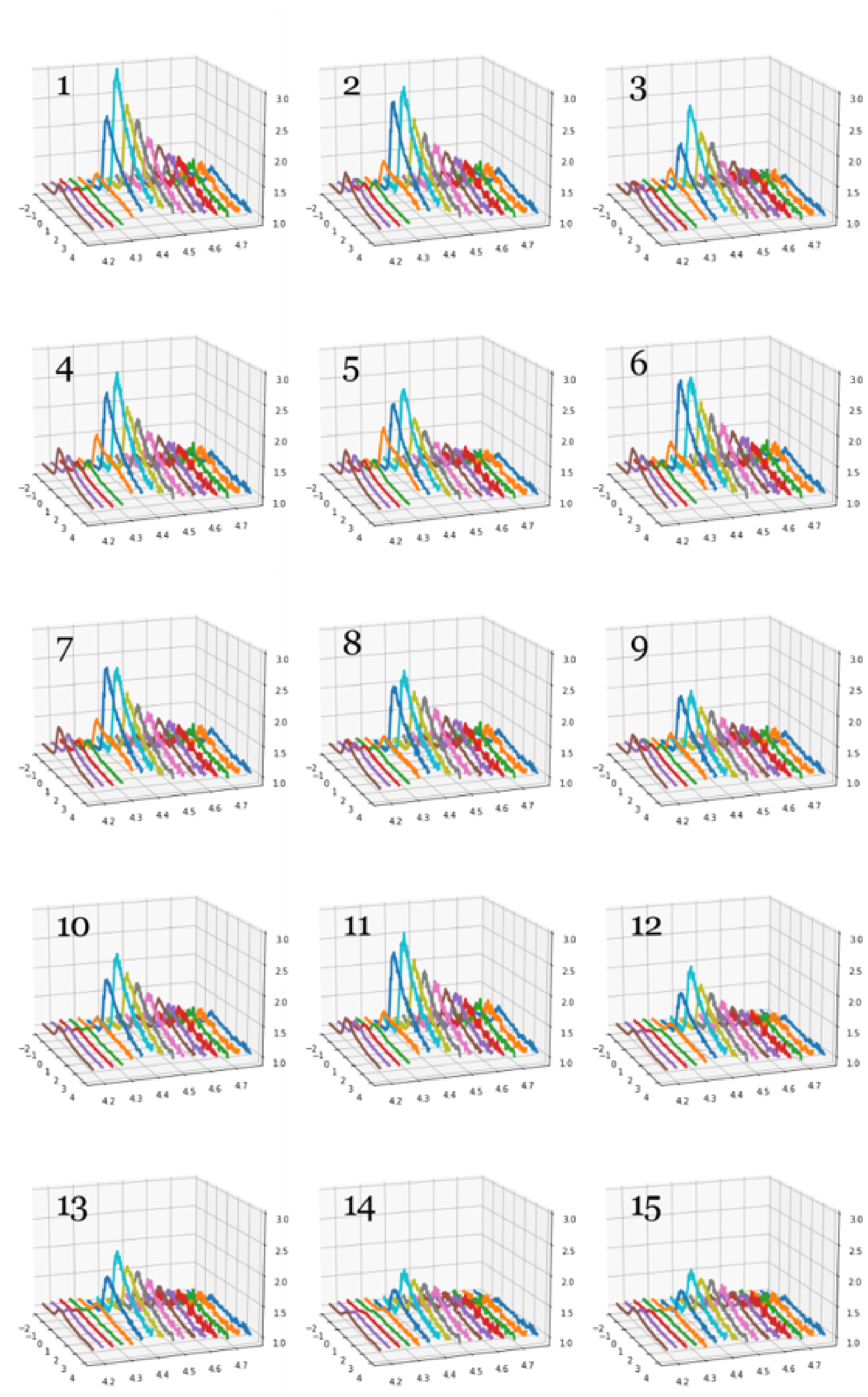

Figure S 2. Time and probe energy dependent PE intensity obtained from all 26 regions. The vertical axis is the ratio of PE intensity with pump irradiation to one with our pump irradiation. The ranges of axes are shown at the bottom. (Figure continues to the next page)

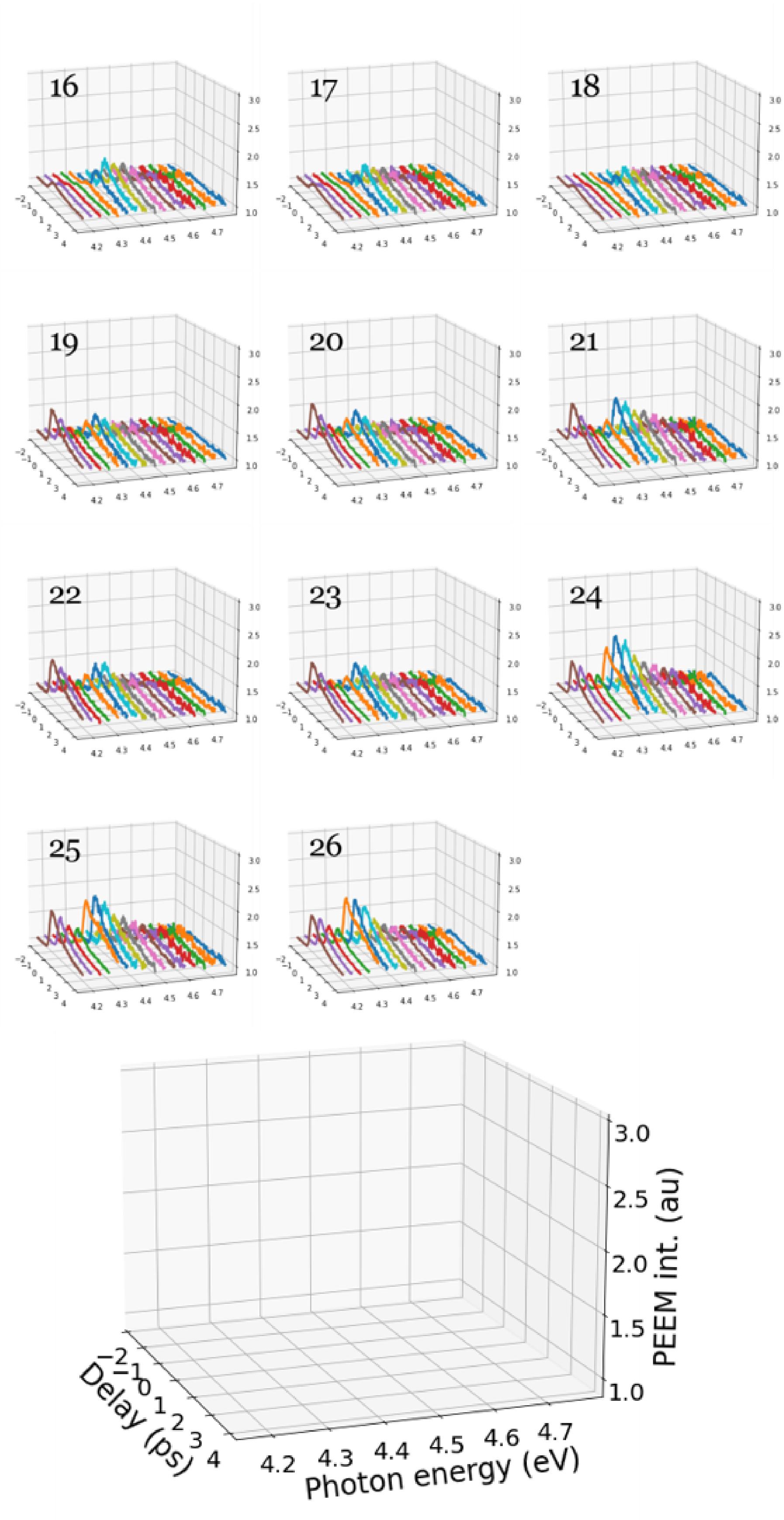
16
17
18
19
20
21
22
23
24
25
26
Delay (ps)
Photon energy (eV)
PEEM int. (au)

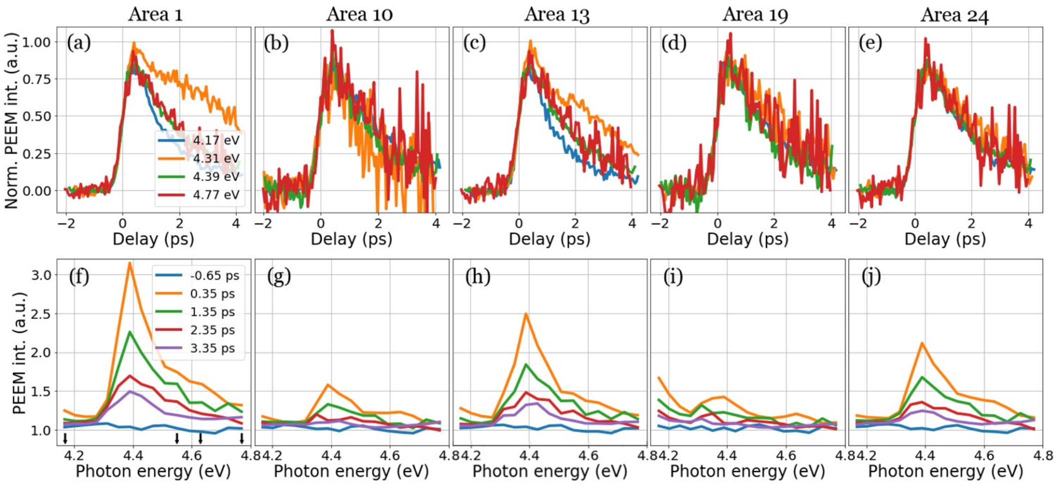

Figure S 3. (a) - (e) Time profiles with different probe energies in regions 1, 10, 13, 19, and 24. (f) - (j) Spectra at different pump-probe delay time in regions 1, 10, 13, 19, and 24.

Selected representative plots were chosen from the five regions (Areas 1, 10, 13, 19 and 24) with probe energies of 4.17, 4.31, 4.39, and 4.77 eV, and are displayed in the upper row of Fig. S**??**, where the vertical axis is normalized from 0 to 1. Although the spectral shapes are similar for the S2 and S4 surfaces, PE intensities below 4.2 eV in Fig. S**??** (i) are more pronounced than in Fig. S**??** (g). Our simulation concludes that the DOS of S4 above the Fermi level is higher than that shown in S2 (Fig. 3 in the main text).

II. SAMPLE PREPARATION

The sample measured was a Bi_2Se_3 single crystal. A surface layer was exfoliated in air and immediately introduced into the ultrahigh vacuum (UHV) chamber to which the photoemission electron microscopy (PEEM) is mounted. To image the surface using PEEM, an electric field as high as 15 kV is applied between the PEEM objective lens and the sample surface with the distance of 2.7 mm to extract photoelectrons (see Figure S**??**). This electric field peels off the surface layers and a clean surface appeared. Figures **??** (a), (b), and (c) show PEEM images taken on the day the sample was introduced, 12 days after, and 15 days after, respectively. One can see that the PEEM contrast changes with time. After 15 days, no further change is observed. Then the CDC–PEEM experiments were performed.

After the CDC–PEEM experiments, the sample was taken out from the UHV chamber,

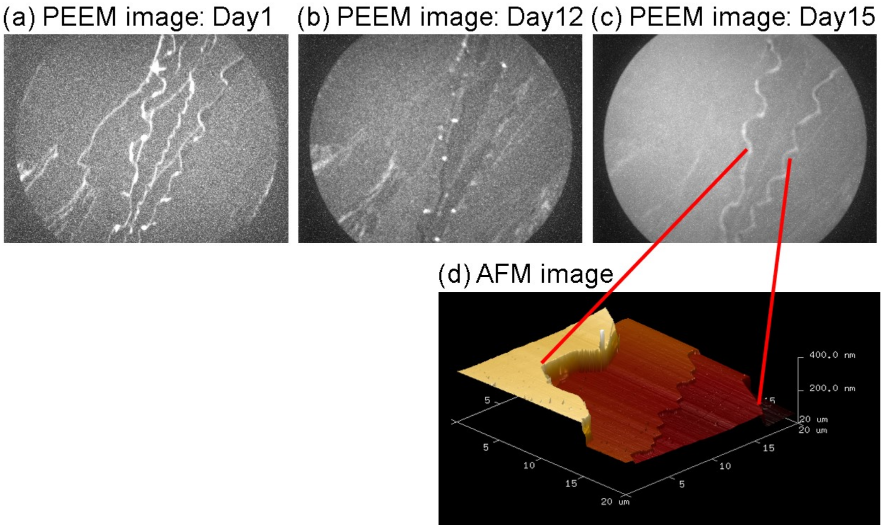

Figure S 4. (a–c) Change of the PEEM images over time due to peeling off the surface layer by electric field between the PEEM objective lens. (d) Comparison of AFM and PEEM images.

and its surface morphology was imaged by atomic force microscopy (AFM) (Figures S?? (d)), and compared with the PEEM images. There is a correlation between the AFM and PEEM images, indicating that the sample surface is exfoliated non-uniformly, which affects the photoemission intensity.

III. OBSERVATION OF SUB-QL STEP HIGHT

AFM experiments were performed in the atmosphere on the same Bi_2Se_3 single crystal measured by CDC-PEEM. The left panel in Figure S5 is an AFM image, and lineprofiles along the red lines named A and B are shown on the right hand side. Step heights are around 1 nm corresponding to one QL-height. In another region, however, step heights were less than one QL-height as seen in Fig. S6. This confirms that Bi_2Se_3 single crystal can be cleaved within QLs.

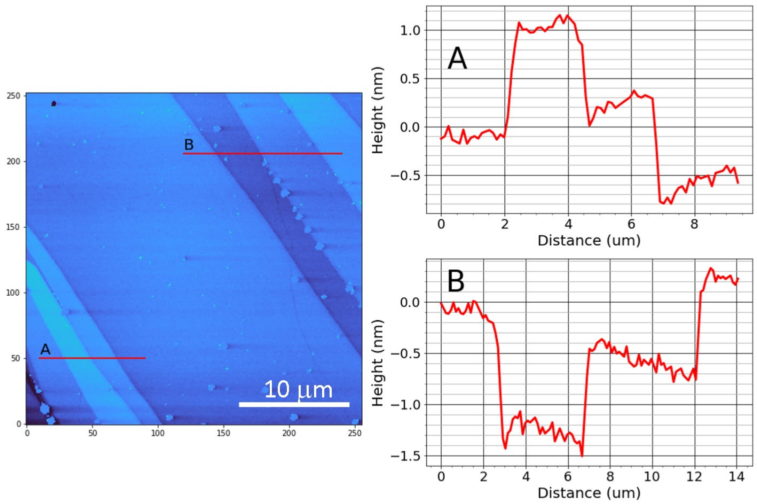

Figure S 5. An AFM image and lineprofiles along the red lines in the image. Step heights are around 1 nm, corresponds to one QL height.

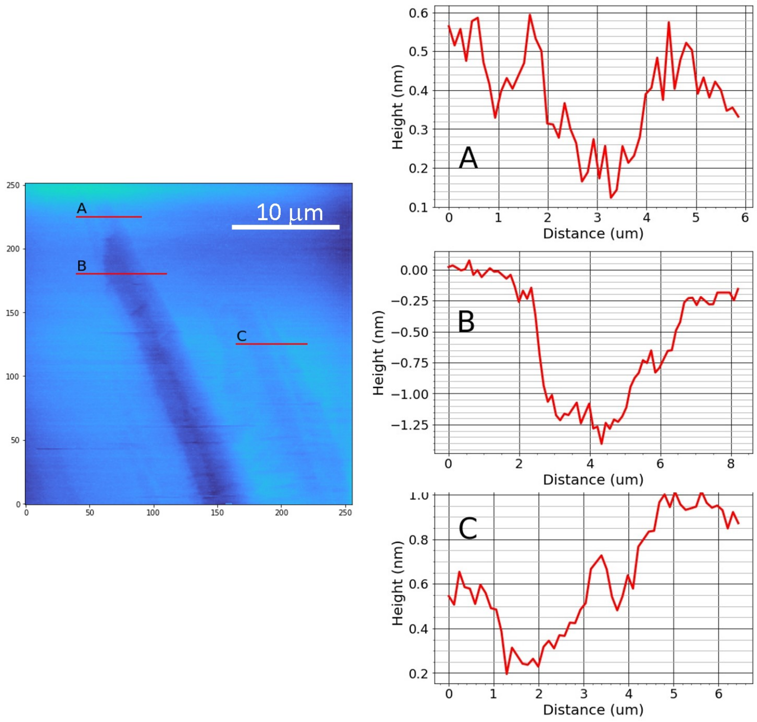

Figure S 6. An AFM image and lineprofiles along the red lines in the image. Step heights are sub-QL heights in this region.
